# Supplementary material for: Reliability of the Polish Version of the Kinesiophobia Causes Scale (KCS) Questionnaire in Assessing the Level of Fear of Movement Among People Suffering from Chronic Nonspecific Low Back Pain
Source: Diagnostics (Basel). 2025 Jul 9;15(14):1746. doi: 10.3390/diagnostics15141746 (PMC12293594; doi:10.3390/diagnostics15141746)
Supplement: Supplementary file 1 [file diagnostics-15-01746-s001.zip › diagnostics-3585811-supplementary.pdf]

Table S1. Results (mean; SD; 95% CI) and intraclass correlation coefficient (ICC) for individual questions of the KCS questionnaire.

Item	Completing of the questionnaire		$ICC_{3.1}$ (95% CI)	SEM
	First (mean ± SD) (95% CI)	After 4 weeks (mean ± SD) (95% CI)		
BD 1	22.77 ±26.8<br>17.76 - 27.78	23.66 ±26.8<br>18.64 - 28.68	0.97<br>(0.96 - 0.98)	0.0
BD 2	31.25 ±23.4<br>26.87 - 35.63	33.26 ±23.1<br>28.93 - 37.58	0.86<br>(0.80 – 0.90)	0.01
BD 3	45.54 ±22.1<br>41.41 - 49.67	48.44 ±21.3<br>44.45 - 52.43	0.82<br>(0.75 – 0.87)	0.2
BD 4	43.97 ±28.7<br>38.59 - 49.35	42.86 ±29.7<br>37.29 - 48.42	0.91<br>(0.87 – 0.93)	0.2
BD 5	42.86 ±34.1<br>36.47 - 49.25	42.86 ±32.1<br>36.85 - 48.87	0.88<br>(0.85 – 0.95)	0.4
BD 6	45.98 ±26.1<br>41.09 - 50.87	46.79 ±25.4<br>42.04 - 51.54	0.89<br>(0.86 – 0.93)	0.1
BD 7	43.30 ±29.6<br>37.76 - 48.85	44.64 ±30.3<br>38.97 - 50.31	0.88<br>(0.84 – 0.92)	0.1
BD 8a	17.86 ±32.8<br>11.72 - 23.99	17.41 ±34.0<br>11.04 - 23.78	0.85<br>(0.79 – 0.89)	0.3
BD 8b	13.39 ±30.7<br>7.64 - 19.15	14.29 ±31.8<br>8.33 - 20.24	0.95<br>(0.93 – 0.97)	0.0
BD 8c	20.98 ±35.3<br>14.38 - 27.59	22.32 ±35.4<br>15.69 - 28.95	0.86<br>(0.81 – 0.91)	0.02
BD 9	24.55 ±27.7<br>19.37 - 29.73	29.02 ±31.9<br>23.04 - 34.99	0.70<br>(0.59 – 0.78)	1.2
BD 10	48.66 ±41.1<br>40.97 - 56.35	44.64 ±39.3<br>37.28 - 52.01	0.87<br>(0.82 – 0.91)	0.02
BD 11	40.63 ±28.1<br>35.36 - 45.89	41.96 ±28.3<br>36.66 - 47.27	0.78<br>(0.69 – 0.84)	0.4
PD 12	41.96 ±44.3<br>33.67 - 50.26	40.63 ±41.1<br>32.93 - 48.32	0.85<br>(0.79 – 0.89)	0.7
PD 13a	26.79 ±44.5<br>18.46 - 35.12	23.21 ±42.4<br>15.27 - 31.16	0.72<br>(0.61 – 0.80)	0.6
PD 13b	30.36 ±46.2<br>21.71 - 39.01	32.14 ±46.9<br>23.36 - 40.93	0.96<br>(0.94 – 0.97)	0.0
PD 13c	33.93 ±47.6<br>25.02 - 42.83	33.93 ±47.6<br>25.02 – 42.83	0.92<br>(0.89 – 0.94)	0.1
PD 14	35.04 ±32.8<br>28.91 - 41.18	35.89 ±31.9<br>29.91 – 41.88	0.85<br>(0.79 – 0.90)	0.3
PD 15	43.97 ±18.8<br>40.46 - 47.49	45.31 ±17.3<br>42.08 - 48.55	0.83<br>(0.77 – 0.88)	0.4
PD 16	55.36 ±35.1<br>48.78 - 61.93	53.13 ±35.7<br>46.44 - 59.81	0.94<br>(0.91 – 0.96)	0.03
PD 17	36.16 ±31.6<br>30.24 - 42.08	33.04 ±31.9<br>27.07 – 39.0	0.86<br>(0.80 – 0.90)	0.1
PD 18	47.77 ±27.2	48.66 ±26.4	0.91	0.02

	42.68 - 52.86	43.72 - 53.60	(0.87 – 0.94)	
PD 19	55.80 ±29.1<br>50.36 - 61.25	58.48 ±29.2<br>53.02 - 63.94	0.87<br>(0.82 – 0.91)	0.02
PD 20	72.77 ±25.0<br>68.09 - 77.45	71.88 ±24.9<br>67.21 - 76.54	0.90<br>(0.85 – 0.93)	0.04

95% CI—95% Confidence Interval; SEM—standard error of measurement; BD—Biological Domain; PD—Psychological Domain. Questions 8 and 13 are three-part questions, hence their designations a, b, c.
